# Some Rays of Hope

**Published:** 1892-02-27

**Authors:** 


### The Hospital

A Weekly Journal of

Science, Medicine, Nursing, and Philanthropy.

*For Hospitals, Asylums, Infirmaries, Dispensaries, Medical Practitioners, Students, Nurses, and the Charitable Public.*

Vol. XI.—No. 283.] [Feb. 27, 1892.

### Some Rays of Hope.

Probably nobody ever considered the Statistical Society a lively association. The other evening the members met to consider a subject which must have been not only dull but depressing. The recent agricultural depression, as it has affected a metropolitan hospital, was dealt with by Dr. J. C. Steele, of Guy's; and Mr. L. L. Price, discussed the same subject in its bearing upon the income of an Oxford College. Dr. Steele was brief and sober; Mr. Price was anything but brief, whilst his sobriety reached a depth which no ordinary mental plummet cares even to attempt to sound. Nevertheless, both the papers were practical and useful, and if one could apply to them a little of the peculiar genius of Mark Tapley, one might extract from them quite a considerable number of rays of comfort and light.

Dr. Steele has held the position of Medical Superintendent at Guy's for close upon forty years. One can easily understand, therefore, why he should consider himself as in some sort *in loco parentis* to the hospital. Its well-being is his well-being; and a dash of melancholy in its fortunes may be expected to show itself in the lines of his face, if not in his coats and gloves. But the doctor is well fitted by nature to fight through a series of depressing circumstances. He is a cautious optimist; the sort of man who, whilst always taking a hopeful view of the situation, steers his bark with the utmost care until it is clear of all the shallows and well out into deep water. Here is a fact in connection with Guy's which might well give check to the most optimistic of medical superintendents. In 1875 the nett income of the hospital from its country estates amounted to £30,919. In 1891 the nett income from the same source reached no more than £17,222, a fall of nearly a half in the course of fifteen years. What has Guy's been doing during those fifteen years of steadily diminishing income? The population of London has been growing, and the demands upon this, as upon all hospitals, have been increasing. But Guy's, instead of lessening its usefulness as its resources have diminished, has not only maintained, but even gone beyond its former standard of indispensable public service.

This expansion, in spite of circumstances that pointed to contraction, has been highly honourable to the responsible managers of Guy's Hospital, and not the least so to Dr. Steele. But it is not to be forgotten that the expansion of Guy's has been secured at the cost of the limitation of some other equally useful medical charities. When the Guy's managers found their resources diminishing, and much of their work threatening to collapse, they boldly and wisely made an appeal to the public for supplementary funds. The response to that appeal constituted some of the rays of light and comfort of which we spoke but a little ago. The public recognised that Guy's had a long and honourable history; that in spite of its being an endowed hospital of independent circumstances it had used its endowments solely for the benefit of the necessitous poor, and for the extension of sound medical knowledge and practice so far as its influence extended. When, therefore, it made its appeal to the charitable for large and immediate funds the response was prompt and generous in a very high degree. Those who take a despairing view of the fortunes of our voluntary hospitals, may study the history of Guy's during the past fifteen years. With an income from landed property that has declined by little and little from £30,919 in 1875 to £17,222 in 1891, this great charitable institution has been able to support an average expenditure of between forty and fifty thousand pounds every year on behalf of the sick and helpless poor. Guy's has, in fact, regarded itself, and with great justice, as a voluntary hospital, and it has prospered accordingly. Its original endowment of £220,000, secured to it in 1724, was a purely voluntary endowment; and the further gift of £193,000 by Mr. William Hunt, of Petersham, in 1829, was also voluntary. It was the most natural thing in the world, then, that when the income from these voluntary sources became so narrowed as to be altogether inadequate for the work, the hospital should, in the spirit of its own constitution, make an immediate appeal to the voluntary liberality of living men.

But what of the future? This is the question which engages the minds of the responsible managers of those voluntary hospitals that have no substantial endowments like those of Guy's to fall back upon in the last resort. Is Guy's to be for ever dipping its hand into the almsdish which constitutes the main supply of the poorer hospitals? We hope not, and it is certain, too, that the Guy's managers themselves hope not. It is exactly here that Mr. Price's contribution to the knowledge of the members of the Statistical Society lends a little confirmation to the cheerful optimism of Dr. Steele. Mr. Price thinks that the agricultural depression, which steadily deepened almost without break from 1875 to 1887, has reached its lowest depth at last. This contention is supported by the experience of an Oxford college, which from a rental of £10,243 in 1876 fell to a rental of £6,498 in 1887. From 1887 to last year there was a steady improvement in the rental of the estates of that particular college. Or, rather, whilst the total actual rent diminished a little the abatements fell from £1,548 in 1887 to £44 in

1890, leaving an increase upon the net rental of nearly a thousand pounds. Those who understand the business will perceive that this fall in the rental abatements from £1,548 to £44 is a fact of the utmost significance. It shows us that the farmer, who, as a rule, is an arrant grumbler, has had so fair a share of prosperity during the past three years that he has practically not ventured to apply to his landlord for any abatement of rent at all.

It is reasonable to assume that what is true of the landed property of an Oxford college will be generally true of all cultivated land throughout the country. And, indeed, we know that this is actually so. Not only is the price of wheat, as of some other agricultural products, higher than it was a few years ago; but other important changes have taken place which tend in the direction of agricultural prosperity. The competition of foreign countries with the producers of this country is somewhat less severe. But that is one of the least considerable of several factors in the case. The two chief circumstances that point to definite and permanent improvement are that the farmers have now at length resolved to adapt themselves to changed circumstances, and that landowners, dismounting from the extremely long-legged horse on which they were wont to ride, have resolved to meet their agricultural tenants half-way, and to give them every possible facility for farming with advantage and profit. This is entirely as it should be. We have the greatest possible faith in both the landlords and the farmers of this country in so far as their practical capacity is concerned. Where both classes fail is in their obstinate adherence to old and impossible positions, and in their resolute determination to do nothing whatever to adjust themselves to new circumstances.

But even an Englishman, be he landowner or be he tenant farmer, finds it disagreeable to run his head against a stone wall oftener than half a dozen times within a strictly limited period. The members of the agricultural interest are now as fully determined to try all the new roads that promise to lead to prosperity as they were formerly resolved not to try any at all. This general determination is of itself an almost certain guarantee of success. It is not likely that agricultural land in this country will, within the next quarter of a century, yield the inflated and artificial rents which it yielded a quarter of a century ago. But there is little reason to doubt that we shall have a return of decided prosperity in agriculture, and that institutions like Guy's Hospital and the Colleges of Oxford, which depend largely for their revenues upon landed property, will be able to carry on their work almost entirely by means of their own ancient resources.

The outlook for the voluntary hospitals is distinctly improved by this turn in the tide of the affairs of agriculture. We have all been surprised at the very small amount contributed by landed proprietors to medical charities during the last dozen or fifteen years. But with returning prosperity those proprietors will have the wherewithal to help our public charities as in former years. The pressure upon merchants, manufacturers, and tradesmen will thus be diminished, and the burden lightened all round.

